# Clinical implications of increased lymph vessel density in the lymphatic metastasis of early-stage invasive cervical carcinoma: a clinical immunohistochemical method study

**DOI:** 10.1186/1471-2407-9-64

**Published:** 2009-02-21

**Authors:** Shi-qian Zhang, Hao Yu, Lin-lin Zhang

**Affiliations:** 1 Department of Gynecology, Qilu Hospital, Shandong University, Jinan 250012, PR China

## Abstract

**Background:**

Cervical cancer is the most common malignant gynecological cancer, and lymphatic metastasis can occur in the early stage of tumor growth. Lymphatic vessel endothelial hyaluronan receptor-1 (LYVE-1), a marker for lymphatic endothelium, provides powerful tools for studying tumor lymphangiogenesis. The purpose of this study is to investigate the clinical implications of lymphangiogenesis in the metastasis of early-stage invasive cervical carcinoma.

**Methods:**

We used immunohistochemical (IHC) staining with the antibody against LYVE-1 to measure lymph vessel density in 41 cases of early-stage invasive cervical carcinoma and 12 cases of normal cervical samples. We then analyzed the correlation between lymph vessel density and clinicopathological features of the tumors.

**Results:**

(1) The majority of peritumoral lymphatics were enlarged, dilated, and irregular. In contrast, intratumoral lymph vessels were small and collapsed. The peritumoral lymphatic vessel density (PLVD) was significantly higher than the intratumoral lymphatic vessel density (ILVD) (*P *< 0.01). (2) Both ILVD and PLVD were significantly higher than the LVD of the control cervixes (*P *< 0.01). (3) Both ILVD and PLVD were significantly associated with lymph node metastasis (ILVD, *P *< 0.05; PLVD, *P *< 0.01) and lymphatic vessel invasion (ILVD, *P *< 0.05; PLVD, *P *< 0.01). Both the ILVD and PLVD in patients with histological grade HG2 and HG3 were significantly higher than those with HG1 (*P *< 0.05).

**Conclusion:**

Tumor lymphangiogenesis in early-stage invasive cervical carcinoma may play an important role in the process of lymphatic metastasis.

## Background

The bloodstream and lymph system are two common pathways by which tumor cells spread. A significant amount of laboratory and clinical research indicates that tumor angiogenesis plays important roles in tumor growth and metastasis. On the other hand, research on tumor lymphangiogenesis lags behind, mostly because of the lack of specific markers of lymphatic endothelium. Recently, several markers for lymphatic endothelium have been discovered, including transmembrane protein lymphatic vessel endothelial hyaluronan receptor-1 (LYVE-1), and these provide powerful tools for studying tumor lymphangiogenesis.

Cervical cancer is the most common malignant gynecological cancer, and lymphatic metastasis can occur in the early stage of tumor growth. The role of tumor lymphangiogenesis in metastasis of early-stage cervical carcinoma is gradually becoming the focus of research. In this study, the role of lymphangiogenesis in cervical cancer metastasis was investigated using clinical patient samples[1].

## Methods

### Patients and tissue samples

Patients (n = 41) with cervical carcinoma who were treated between September 2007 and June 2008 were enrolled in the study. The tissue samples were obtained at the time of surgery in the Department of Gynecology, Qilu Hospital, Shandong University. The present study was approved by the ethics committee of the Shandong University. All samples were obtained with medical-ethics approval and all patients gave informed consent. Tissues were fixed with 4% paraformaldehyde and paraffin-embedded for further analysis. The pathological examination validated the fact that no radio- or chemotherapy was received before surgery. All patients with early-stage invasive cervical cancer were staged according to the 2000 International Federation of Gynecology and Obstetrics (FIGO) staging system. Seven patients had a cervical cancer classified as FIGO stage Ia, 14 as FIGO stage Ib, and 20 as FIGO stage IIa. Based on analysis of cellular differentiation, seven cases were HG1, 13 were HG2, and 21 were HG3. Of all the cases, 37 were squamous cell carcinomas and four were adeno-carcinomas. Histological review confirmed that 15 cases showed lymph node metastasis. Lymph node status was determined by routine pathological examination with the surgical specimens. The standard for lymphatic vessel invasion was the detection of cancer cells in the cavity of the lymphatic vessel by light microscopy. By this standard, 35 cases showed lymphatic vessel invasion and six were negative. The age of the patients varied from 26 to 70, with a median value of 42. Of all the patients, 29 were premenopausal and 16 were postmenopausal. Pelvic/paraaortic lymph node dissection was performed in all cases; the mean and median number of lymph nodes per case examined was 24.7 and 24.0, respectively (range 1–82). Lymph node status was determined by routine pathological examination with the surgical specimens. Lymph node metastasis was present in 15 (36.6%) cases; the mean and median number of positive lymph nodes was 2.5 and 2.0, respectively (range 1–7). All tissue specimens and slides were examined by experienced pathologists.

### Immunohistochemistry

Immunohistochemistry (IHC) was done using the immunohistochemistry SP kit from Jingmei Inc. (Shanghai, China; catalog no. LHK612) according to the manufacturer's instructions. Briefly, serial section slides of 5 μm were obtained from the paraffin-embedded specimens and the paraffin medium was removed. The slides were then re-hydrated by passing through a descending serial alcohol dilutions. Then the slides were placed in antigen retrieval solution (pH 6.0) and heated in a microwave oven for 10 min at 95°C. Next, the slides were incubated in a 3% hydrogen peroxide-methanol solution for 10 min to remove endogenous peroxidase. IHC staining was performed as follows: nonspecific binding was blocked with 10% goat serum; the slide was incubated for 1 h with polyclonal rabbit anti-LYVE-1 antibody (1:80 dilution, Abcam, catalog # ab36993), followed by incubation for 10 min with biotin-labeled secondary antibody; then the slide was incubated for 10 min with horseradish peroxidase-labeled streptavidin. Color was developed using DAB, and the slide was counterstained with hematoxylin. Finally, the slides were mounted and coverslipped with resinene. Negative control slides were stained with PBS instead of LYVE-1 antibody.

Intratumoral and peritumoral lymphatic vessel density (LVD) was determined according to the methods previously described by Weidner et al.[2] and Gombos et al.[3]. Briefly, the following had to be observed in order for lymphatic vessels to be considered positively stained: (1) brown-yellow stained endothelial cells forming tubular structures and situated on their own or bundled, and (2) an open cavity inside the tubular structure. The slides were scanned on a low-power microscope and intra- and peritumoral areas with the highest vessel density, called hot spots, were identified. The number of lymphatic vessels in five high-power fields in the selected areas was counted. LVD was determined as the mean value of vessel counts.

Intratumoral lymphatic vessels were defined as those located within the tumor mass. Peritumoral lymphatic vessels were those located outside of the tumor mass, but within 2 mm from the tumor. The lymphatic vessels in the control samples were defined as those located in the stromal area, within 2 mm of the underlying basement membranes. The slides were evaluated independently by investigators blinded to the clinical data.

### Statistical Analysis

All statistical calculations were performed using SPSS (version 13.0, Chicago, IL USA). LVDs are expressed as mean ± SD. The statistical methods used include the t-test, one-way ANOVA test, and the Kruskal-Wallis test. Differences were considered statistically significant when *P *< 0.05.

## Results

### Morphology of lymphatic vessels in cervical cancer tissue

**Figure 1:**
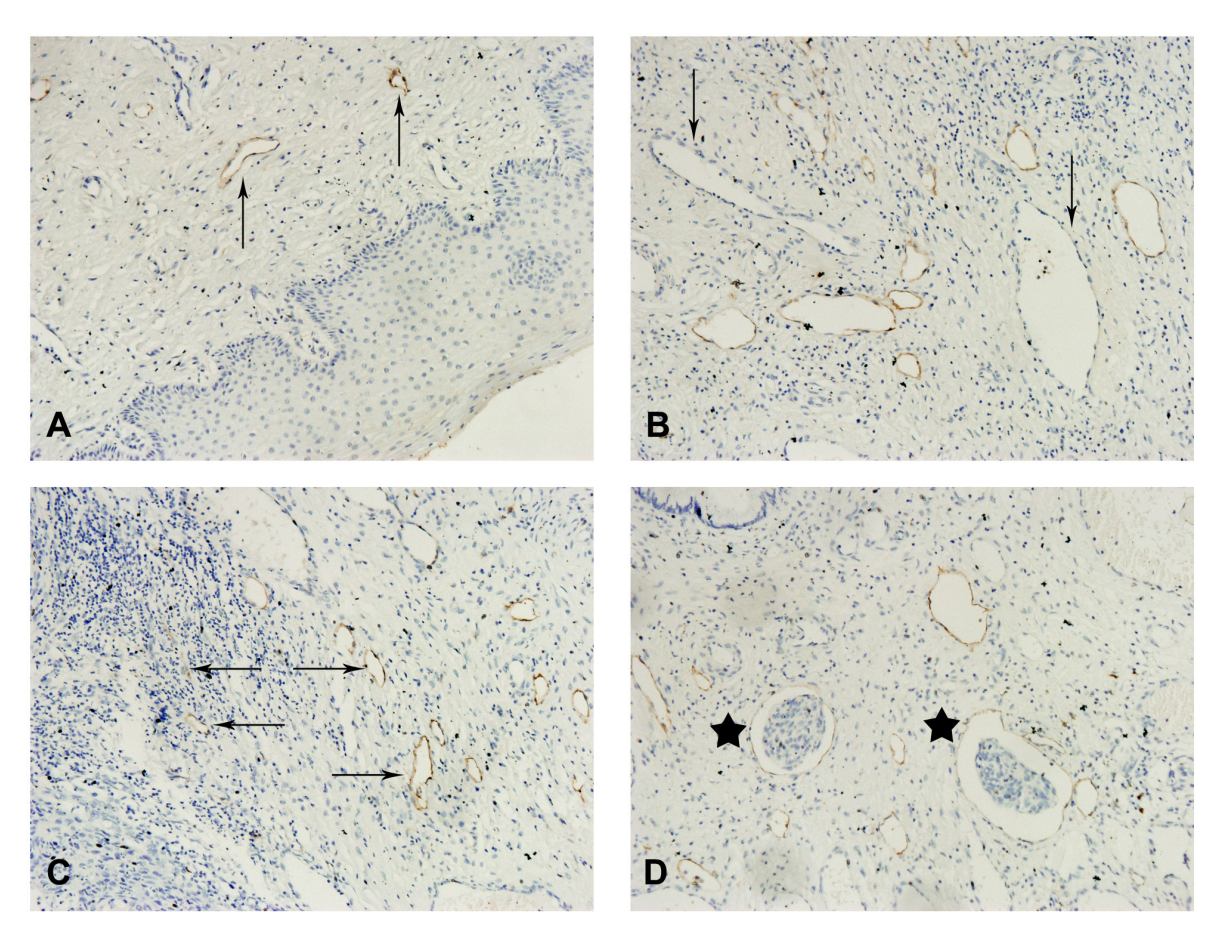
**Morphology of lymphatic vessels in cervical cancer tissue**. (A) The lymph vessels of the normal cervix control were open and regular (arrow). Primary antibody: LYVE-1 polyclonal antibody, magnification: ×200. (B) Blood vessel endothelial cells, negative by immunostaining, with some red blood cells in the capillary lumen (arrow). Primary antibody: LYVE-1 polyclonal antibody, magnification: ×400. (C) The intratumoral lymph vessels were small and collapsed (←); in contrast, the peritumoral lymphatics were often dilated, and irregularly shaped (→). Primary antibody: LYVE-1 polyclonal antibody, magnification: ×200. (D) Some invading cancer cells were present in lymph vessels (star). Primary antibody: LYVE-1 polyclonal antibody, magnification: ×400.

Lymphatic vessels in the control uterine cervix are often open and have a regular shape (Figure 1A). Under the high-power light microscope, the positively stained vessels were brown-yellow and the vessel walls were thin. The vessel endothelium was usually a single layer of cells, each with a large nucleus extruding toward the lumen face (Figure 1B). In contrast, most of the lymphatic vessels in cervical carcinoma tissue were localized around the tumor mass in increased numbers. They were often in irregular shape with an enlarged cavity. The lymphatic vessels in the tumor mass were small, collapsed tubular structures that were either isolated or bundled (Figure 1C). At the same time, invasive cancer cells were observed in some peritumoral lymphatic vessels (Figure 1D).

### Lymphatic vessel density

The intratumoral lymphatic vessel density (ILVD) was 7.71 ± 1.12 and peritumoral lymphatic vessel density (PLVD) was 9.10 ± 0.80. PLVD was significantly higher than ILVD (*P *< 0.01) in the corresponding cervical carcinoma samples. The ILVD and PLVD associated with carcinomas *in situ *were both significantly higher than the LVD of the normal uterine cervix (2.08 ± 1.00) (*P *< 0.01).

The clinicopathological features and LVD data are summarized in Table 1. In samples from patients with lymph node metastasis, the ILVD was 8.27 ± 0.80 and the PLVD was 9.67 ± 0.72. Both of these values were significantly higher than the corresponding values in the samples without metastasis (7.38 ± 1.17, 8.77 ± 0.65, respectively; *P *< 0.05, *P *< 0.01). Similarly, ILVD and PLVD were found to correlate significantly with lymphatic vessel invasion (*P *< 0.05 and *P *< 0.01, respectively). The ILVD and PLVD from cases showing lymphatic vessel invasion were much higher than in those cases without invasion although these differences did not reach statistical significance (8.67 ± 0.82 vs. 7.54 ± 1.09, 9.67 ± 0.72 vs. 8.94 ± 0.73).

**Table 1:** Association of LVD and other clinical and pathological parameters. P, t-test; P*, one-way ANOVA test; P**, Kruskal-Wallis test.

		ILVD		PLVD	
			
Variables	*N*	mean ± SD	*P*	mean ± SD	*P*
Catamenia					
Premenopause	29	7.83 ± 1.10	0.292	9.17 ± 0.80	0.358
Postmenopause	12	7.42 ± 1.12		8.92 ± 0.79	
Tumor size (cm)					
≤ 4	26	7.58 ± 1.06	0.528	9.08 ± 0.84	0.831
> 4	15	7.93 ± 1.22		9.13 ± 0.74	
Stromal invasion					
≤ 2/3	17	7.35 ± 0.86	0.089	8.94 ± 0.75	0.298
> 2/3	24	7.96 ± 1.23		9.21 ± 0.83	
FIGO stage					
Ia	7	6.86 ± 1.07	0.075*	8.57 ± 0.79	0.113*
Ib	14	8.00 ± 1.24		9.07 ± 0.62	
IIa	20	7.80 ± 0.21		9.30 ± 0.86	
Histological grade					
HG_1_	7	6.57 ± 0.96	0.009*	8.29 ± 0.76	0.012**
HG_2_	13	7.85 ± 1.14		9.31 ± 0.63	
HG_3_	21	8.00 ± 0.95		9.24 ± 0.77	
Lymph node metastasis					
Negative	26	7.38 ± 1.17	0.013	8.77 ± 0.65	0.0002
Positive	15	8.27 ± 0.80		9.67 ± 0.72	
LVI					
Negative	35	7.54 ± 1.09	0.021	8.94 ± 0.73	0.001
Positive	6	8.67 ± 0.82		10.00 ± 0.63	
Histological cell type					
SCC	37	7.62 ± 1.14	0.139	9.14 ± 0.79	0.367
ADE	4	8.50 ± 0.58		8.75 ± 0.96	

Abbreviation: HG, histological grade; LVI, lymphatic vessel invasion; SCC, squamous cell carcinoma; ADE, adenocarcinoma.

Meanwhile, we also analyzed the correlation between the histological grade and the ILVD or PLVD. The ILVDs for cases of HG1, HG2, and HG3 were 6.57 ± 0.96, 7.85 ± 1.14, and 8.00 ± 0.95, respectively. The PLVDs for cases of HG1, HG2, and HG3 were 8.29 ± 0.76, 9.31 ± 0.63, and 9.24 ± 0.77, respectively. The ILVDs and PLVDs for HG2 and HG3 cases were significantly higher than the corresponding values for HG1 cases (*P *< 0.05). There was no significant difference between the HG2 and HG3 cases. On the other hand, LVDs showed no correlation with patient clinical stages, menopause condition, or carcinoma histology. The depth of tumor invasion, an important clinicopathological feature, was not correlation with depth of tumor invasion and the lymph vessel density.

## Discussion

The lymphatic system is involved in multiple physiological processes, such as maintenance of the internal environment and immune surveillance. It also plays important roles in different kinds of pathological processes, including lymphatic swelling, tissue repair, inflammation, and especially tumor metastasis[4]. The last process is a major risk factor for tumor prognosis. Cervical cancer metastasizes mostly by direct spreading and lymphatic invasion, but rarely by traveling through the bloodstream. Therefore, studying lymphangiogenesis has important implications for understanding lymphatic metastasis of cervical cancer. Currently, the available endothelial markers for lymphatic vessels include vascular endothelial growth factor receptor-3 (VEGFR-3), LYVE-1, Podoplanin, Prox1, and others. LYVE-1 is the homologue of CD44, and its main physiological functions include serving as a receptor for hyaluronic acid (HA) and facilitating the transport and metabolism of HA in the extracellular matrix. It has also been shown that LYVE-1 is involved in adherence of tumor cells[5]. LYVE-1 is distributed evenly on the basal and luminal surfaces of lymphatic vessels and is considered to be the most useful marker for lymphatic endothelium[6].

In the current study, we used a polyclonal LYVE-1 antibody to label lymphatic vessels. We found that positively stained lymphatic vessels had an enlarged lumen lacking red blood cells. Some invasive cancer cells were observed inside the lumen. Their walls were often irregular and built up of a single layer of endothelial cells, each with a large, extruding nucleus. In contrast, the walls of blood vessels were thick, regular, and consisted of endothelial cells with flattened nuclei. The lymphatic vessels were easily distinguished from blood vessels, because the latter always contained a large amount of red blood cells. In addition, we found lymphatic vessels in cervical cancers to differ morphologically from lymphatic vessels in a normal cervix. The lymphatic vessels in the control cases were open and regular. The corresponding vessels in cancer specimens, however, were more numerous and unevenly distributed, showing a higher density in the peritumoral area. The morphology of peritumoral lymphatic vessels was usually dilated and irregular, while the lymphatic vessels in tumor masses were often closed and flattened. These results are consistent with previous studies[7]. A possible explanation for this finding is that the tumor mass increases so quickly, due to rapid cell proliferation, that the newly developed lymphatic vessels are under high stromal hydrostatic pressure. Tumor cell invasion may also impair the integrity of lymphatic vessels[8].

The idea that tumors induce growth of new lymphatic vessels has been controversial. Recently, there have been significant improvements in lymphatic endothelial cell growth factors, markers, and isolation techniques. Using these new techniques, a large number of animal and clinical studies have confirmed the existence of a newly developed lymphatic network in the body of a tumor. In animal tumor models with high-expression levels of lymphatic vessel growth factor VEGF-C[9] or VEGF-D[10], the endothelial cells along lymphatic vessels show proliferation and a positive stain for proliferating cell nuclear antigen (PCNA). The ILVD and PLVD in these tumors are also higher than those in normal tissues. In our study, we found that the ILVD and PLVD of cervical carcinomas are significantly higher than those of normal cervix tissues. This is consistent with other clinical studies[11,12]. Under the regulation of lymphatic vessel growth factors, lymphatic endothelial cells undergo proliferation and migration to form new vessel cavities. The newly developed tumor lymphatic vessels have a structure similar to that of lymphatic vessels under normal physiological conditions, but they are more numerous and they have thin, leaky walls. These features facilitate the metastasis of tumor cells.

Thus far, most studies suggest that the lymphatic network in the peritumoral area may play an important role in tumor metastasis[13]. Padera *et al*. performed a dye absorption experiment and showed that most of the functional lymphatic vessels in tumors are located around the tumor mass and are positive for PCNA expression[14]. There is some debate about whether the new lymphatic vessels in the tumor body are functional. Clinical research has revealed that although the lymphatic vessels in the tumor mass are PCNA-positive, they may not be functional since they usually consist of a low number of small and flattened endothelial cells. However, studies using animal models featuring a high level of lymphatic growth factors show that new lymphatic vessels strongly correlate with lymph node metastasis of tumor cells[15]. Therefore, the newly growing lymphatic vessels have been hypothesized to lie within the tumor mass. As the tumor cells proliferate, the stromal hydrostatic pressure increases rapidly and leads to the blockage of intratumoral lymphatic vessels. Consistent with Gombos *et al*.[3], we have shown here that the ILVD and PLVD strongly correlate with lymph node metastasis and lymphatic vessel invasion. In summary, both peri- and intratumoral lymphatic vessels are involved in lymphatic invasion and metastasis of tumor cells. Intratumoral lymphatic vessels may function to provide a direct pathway for the tumor cell to metastasize to the lymph nodes in the early stage of tumor growth, when the stromal hydrostatic pressure is not high enough to block them. Another possible mechanism is the more numerous and dilated peritumoral lymphatic vessels may serve as the metastasis pathway. During this process, LYVE-1 molecules may provide adhesive force for tumor cells and further promote tumor lymphatic invasion and lymph node metastasis.

We found that the ILVD and PLVD correlate with histological grade. The ILVD and PLVD of medium-low differentiated specimens were significantly higher than those of the highly differentiated samples. The reason for this may be that the medium-low differentiated tumor cells are more malignant and can secrete more growth factors that induce lymphatic vessel formation and lymph node metastasis. In addition, we also showed that ILVD and PLVD do not correlate with patient clinical staging, menopause state, or histological type.

## Conclusion

The LVD of early-stage cervical carcinoma is significantly higher than that of a normal cervix, and it strongly correlates with multiple clinicopathological features, such as lymph node metastasis, lymph vessel invasion, and histological grade. Therefore, lymphangiogenesis may play an important role in lymphatic vessel metastasis of early-stage cervical carcinoma.

## Competing interests

The authors declare that they have no competing interests.

## Authors' contributions

SZ was the guarantor of integrity of the entire study and designed the experiment, carried out the immunohistochemical method studies and drafted the manuscript. HY participated in literature research, data analysis and manuscript editing. LZ designed the experiment, carried out the immunohistochemical method studies and participated in manuscript preparation. All authors read and approved the final manuscript.

## Pre-publication history

The pre-publication history for this paper can be accessed here:

http://www.biomedcentral.com/1471-2407/9/64/prepub

## References

[B1] Ji RC Lymphatic endothelial cells, tumor lymphangiogenesis and metastasis: New insights into intratumoral and peritumoral lymphatics Cancer Metastasis Rev 2006 25 677 694 10.1007/s10555-006-9026-y 17160713

[B2] Weidner N Semple JP Welch WR Folkman J Tumor angiogenesis and metastasis – correlation in invasive breast carcinoma N Engl J Med 1991 324 1 8 1701519 10.1056/NEJM199101033240101

[B3] Gombos Z Xu X Chu CS Zhang PJ Acs G Peritumoral lymphatic vessel density and vascular endothelial growth factor C expression in early-stage squamous cell carcinoma of the uterine cervix Clin Cancer Res 2005 11 8364 8371 10.1158/1078-0432.CCR-05-1238 16322297

[B4] Tobler NE Detmar M Tumor and lymph node lymphangiogenesis – impact on cancer metastasis J Leukoc Biol 2006 80 691 696 10.1189/jlb.1105653 16793912

[B5] Jackson DG Prevo R Clasper S Banerji S LYVE-1, the lymphatic system and tumor lymphangiogenesis Trends Immunol 2001 22 317 321 10.1016/S1471-4906(01)01936-6 11377291

[B6] Jackson DG Biology of the lymphatic marker LYVE-1 and applications in research into lymphatic trafficking and lymphangiogenesis APMIS 2004 112 526 538 10.1111/j.1600-0463.2004.apm11207-0811.x 15563314

[B7] Zhang JP Lu WG Chen HZ Zhou CY Xie X [Tumor lymphangiogenesis in cervical squamous cell carcinoma and its clinical significance] Zhonghua Yi Xue Za Zhi 2005 85 1551 1554 16179117

[B8] Kato S Shimoda H Ji RC Miura M Lymphangiogenesis and expression of specific molecules as lymphatic endothelial cell markers Anat Sci Int 2006 81 71 83 10.1111/j.1447-073X.2006.00142.x 16800291

[B9] Jennbacken K Vallbo C Wang W Damber JE Expression of vascular endothelial growth factor C (VEGF-C) and VEGF receptor-3 in human prostate cancer is associated with regional lymph node metastasis Prostate 2005 65 110 116 10.1002/pros.20276 15880525

[B10] Kopfstein L Veikkola T Djonov VG Baeriswyl V Schomber T Strittmatter K Stacker SA Achen MG Alitalo K Christofori G Distinct roles of vascular endothelial growth factor-D in lymphangiogenesis and metastasis Am J Pathol 2007 170 1348 1361 17392173 10.2353/ajpath.2007.060835 PMC1829467

[B11] Gao P Zhou GY Zhang QH Xiang L Zhang SL Li C Sun YL Clinicopathological significance of peritumoral lymphatic vessel density in gastric carcinoma Cancer Lett 2008 263 223 230 10.1016/j.canlet.2008.01.017 18289774

[B12] Longatto-Filho A Pinheiro C Pereira SM Etlinger D Moreira MA Jube LF Queiroz GS Baltazar F Schmitt FC Lymphatic vessel density and epithelial D2-40 immunoreactivity in pre-invasive and invasive lesions of the uterine cervix Gynecol Oncol 2007 107 45 51 10.1016/j.ygyno.2007.05.029 17604828

[B13] Ji RC Lymphatic endothelial cells, tumor lymphangiogenesis and metastasis: New insights into intratumoral and peritumoral lymphatics Cancer Metastasis Rev 2006 25 677 694 10.1007/s10555-006-9026-y 17160713

[B14] Padera TP Kadambi A di Tomaso E Carreira CM Brown EB Boucher Y Choi NC Mathisen D Wain J Mark EJ Lymphatic metastasis in the absence of functional intratumor lymphatics Science 2002 296 1883 1886 10.1126/science.1071420 11976409

[B15] Franchi A Massi D Santucci M Masini E Degl'Innocenti DR Magnelli L Fanti E Naldini A Ardinghi C Carraro F Inducible nitric oxide synthase activity correlates with lymphangiogenesis and vascular endothelial growth factor-C expression in head and neck squamous cell carcinoma J Pathol 2006 208 439 445 10.1002/path.1892 16278821

